# Determining the duration of an ultra-intense laser pulse directly in its focus

**DOI:** 10.1038/s41598-019-55949-3

**Published:** 2019-12-20

**Authors:** Felix Mackenroth, Amol R. Holkundkar

**Affiliations:** 1 Max Planck Institute for the Physics of Complex Systems, Dresden, Germany; 2 Department of Physics, Birla Institute of Technology and Science, Pilani, Rajasthan 333031 India

**Keywords:** High-field lasers, Nonlinear optics, Characterization and analytical techniques

## Abstract

Ultra-intense lasers facilitate studies of matter and particle dynamics at unprecedented electromagnetic field strengths. In order to quantify these studies, precise knowledge of the laser’s spatiotemporal shape is required. Due to material damage, however, conventional metrology devices are inapplicable at highest intensities, limiting laser metrology there to indirect schemes at attenuated intensities. Direct metrology, capable of benchmarking these methods, thus far only provides static properties of short-pulsed lasers with no scheme suggested to extract dynamical laser properties. Most notably, this leaves an ultra-intense laser pulse’s duration in its focus unknown at full intensity. Here we demonstrate how the electromagnetic radiation pattern emitted by an electron bunch with a temporal energy chirp colliding with the laser pulse depends on the laser’s pulse duration. This could eventually facilitate to determine the pulse’s temporal duration directly in its focus at full intensity, in an example case to an accuracy of order 10% for fs-pulses, indicating the possibility of an order-of magnitude estimation of this previously inaccessible parameter.

## Introduction

## Ultra-intense Laser Metrology

The development of lasers towards ever higher intensities, motivated by their utility as tools for studies of fundamental physics^1,2^ and applications such as particle acceleration^3^ or high-energy photon sources^4,5^, is driven by compressing their energy to ever shorter pulse durations. Several high-power facilities are already operating pulse durations of less than 30 fs^6–9^, with facilities under construction, aiming at pulse energies exceeding 100 J at less than 10 fs pulse durations^10–13^. In this parameter regime, novel physics are predicted or were readily observed, ranging from the broadening of emission spectra^14^ to the sub-cycle dependence of atomic ionization^15^ or even electron-positron pair production^16,17^. All these effects feature a delicate dependence on the laser’s spatial and temporal profiles. These, however, may distort unpredictably upon amplification and propagation^18,19^. Consequently, a thorough characterization of the laser field inside its focus, where the interaction takes place, is required. Due to material damage, on the other hand^20^, ultra-intense laser pulses cannot be sent directly through solid state devices, conventionally used to measure pulse durations^21–24^, energies^25^, spot sizes^26^ and sub-cycle field structures^15,27–32^ at lower intensities. The strong electromagnetic fields of ultra-intense lasers as considered in this work would immediately disintegrate such devices whence metrology has to be performed either far from focus or at attenuated intensities. On the other hand, attenuating the laser’s full intensity or using a secondary laser beam line of reduced intensity would amount to using indirect pulse characterization schemes. Such indirect schemes, however, should, at least in principle, be benchmarked by direct measurements, providing a signal originating directly from the unattenuated and fully focused laser pulse. The desire for direct laser characterization techniques, benchmarking the well-established indirect methods was consequently expressed in community meetings^33^ as well as by experts on experimental high-power laser science^34^. One possible solution is quantifying the emission patterns of electrons scattered from the laser focus, giving access to a direct determination of its carrier-envelope phase^35,36^ and intensity^18^, with the latter already implemented experimentally^37^. This latter experiment is in line with a series of recent experiments on laser-electron accelerators^38–40^ and all-optical radiation sources^4,5,41–43^, for which there exists an abundance of refined detectors for both the electrons and emitted radiation^44^. These schemes are based on the interaction of electrons (mass and charge *m* and *e* < 0, respectively) with initial momenta ${p}_{i}^{\mu }=({\varepsilon }_{i}/c,{{\boldsymbol{p}}}_{i})$, where *c* is the speed of light, with an ultra-intense laser pulse of peak electric field *E*_0_ and central frequency *ω*_0_, corresponding to a wavelength ${\lambda }_{0}=2\pi c/{\omega }_{0}$. Here, ultra-intense refers to lasers for which the dimensionless amplitude

1 $$\xi =\frac{|e|{E}_{0}}{{\omega }_{0}mc}$$

exceeds unity, indicating that the electrons will be accelerated to relativistic velocities within one field oscillation. A relativistic electron ${\varepsilon }_{i}\gg m{c}^{2}$, scattered from an ultra-intense laser pulse $\xi \gg 1$, emits radiation into a cone around its or the laser’s initial propagation direction with opening angle^35,45^

2 $$\delta \zeta \sim \{\begin{array}{cc}\frac{m{c}^{2}\xi }{{\varepsilon }_{i}} & {\rm{i}}{\rm{f}}\,{\varepsilon }_{i}\gg m{c}^{2}\xi \\ \frac{{\varepsilon }_{i}}{m{c}^{2}\xi } & {\rm{i}}{\rm{f}}\,{\varepsilon }_{i}\ll m{c}^{2}\xi \\ 1 & {\rm{i}}{\rm{f}}\,{\varepsilon }_{i}\approx m{c}^{2}\xi .\end{array}$$

On the other hand, the pulse duration, a highly relevant pulse parameter, cannot be determined at full intensity by either solid state devices or the electron scattering schemes mentioned above, indicating the lack of a possibility to benchmark indirect pulse duration measurements in ultra-intense lasers’ foci at full intensity.

Here we present a fundamentally novel approach to amend well-developed pulse metrology techniques operating at attenuated intensities. We show how an ultra-intense laser’s pulse duration can in principle be measured directly in its focus at ultra-high field strengths, providing an order-of-magnitude benchmark for conventional indirect pulse metrology. Its basic working principle is to imprint a temporal structure onto the electron bunch to be scattered from the ultra-intense laser pulse, in the form of an energy chirp as are common in laser-accelerated electron bunches and need to be mitigated with great effort^46,47^. Keeping the electron bunch chirped, the particles’ changing energy will lead to a temporal evolution of the angular range into which the electrons emit radiation. Here we quantify this temporal change by means of a simplified analytical model which is then benchmarked by numerical simulations, demonstrating that the pulse’s duration can be quantitatively inferred. To assess how feasible the proposed scheme is we corroborate our analysis by a thorough analysis of the relevant error sources affecting its experimental implementation.

## Analytical Model

**Figure 1:**
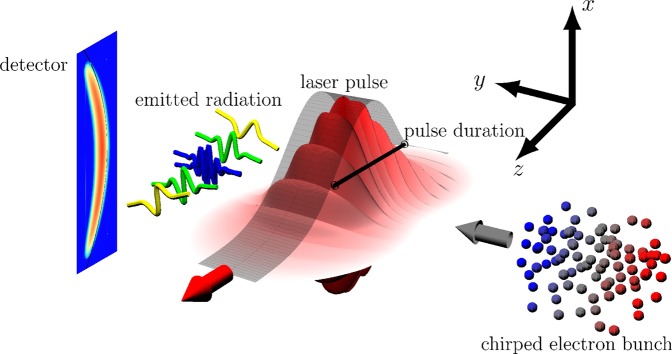
Schematic view of the proposed pulse duration measurement scheme and used coordinate frame.

**Figure 2:**
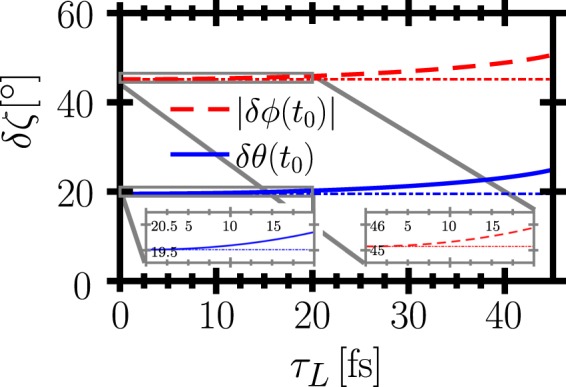
Theoretical predictions for the electrons’ maximal angular deflections from their initial propagation direction as functions of the pulse duration. For comparison we show the maximum deflection angles for an unchirped electron bunch (thin dashdotted lines). The inlays show zoom-ins on the short-pulse behavior of $\delta \theta ({t}_{0}),\delta \phi ({t}_{0})$.

To extract the electrons’ radiation signal from the laser’s focal region and to decouple it from the strong optical laser radiation background, it is favorable to let the electrons collide with the laser pulse perpendicularly. We thus analyze the emission from an electron bunch propagating along the *y*-axis colliding with a laser pulse propagating along the positive *z*-axis and polarized along the *x*-axis (s. Fig. 1). In order to quantify the overall angular distribution of the bunch’s emitted radiation, we observe that at each time instant the radiation emitted by an ultra-relativistic electron is confined to a narrow cone around its instantaneous direction of propagation. Thus, we approximate the time-dependent direction into which radiation is emitted as the time-dependent electron propagation direction. Naturally, this approximation is limited for low-energy electrons, but from a numerical simulation, taking into account the exact angular distribution of an electron’s emission, we find it to be still reliable for energies as low as 5 MeV (s. below). We thus need to solve an electron’s equation of motion, which is generally involved in a laser field of arbitrary focusing. On the other hand, it was demonstrated that for schemes utilizing the emission of a laser-driven electron bunch a clear detection signal is provided by the boundaries of the angular radiation distribution^18,35,37^, determined by the strongest electron deflection, i.e., at the regions of highest field strength. Consequently, we focus on the electron dynamics close to the laser’s focus, which we assume to lie in the origin $(x,y,z)\equiv {\bf{0}}$ in the following. In this focal region the laser field is well approximated by a plane wave, provided the transit time of the electrons through the focal volume *τ*_*t*_ is longer than the pulse duration *τ*_*L*_, so that they will not experience non-plane wave field contributions far away from the laser focus. The ratio of these quantities, which has to exceed unity, can be found as ${\tau }_{t}/{\tau }_{L}=2{w}_{0}/(c{\tau }_{L})$, where *w*_0_ is the laser’s spot radius and we assumed the electrons to propagate with the speed of light. We thus see that the plane wave approximation is valid only for not too tight focusing and correspondingly short laser pulses. It will be demonstrated, however, that for FWHM pulse durations down to *τ*_*L*_ ~ 10 fs the analytical model provides good agreement with a full numerical simulation. We assume the above condition to be satisfied and model the laser field as a plane wave with four-potential ${A}^{\mu }(\eta )=(m{c}^{2}\xi /|e|){\varepsilon }_{0}^{\mu }g(\eta )$, with $\eta =t-z/c$, the pulse’s polarization vector ${\varepsilon }_{0}^{\mu }$ and temporal shape *g*(*η*). We quantify the electrons’ emission direction by the spherical coordinate angles *θ* and *ϕ*, with respect to the *z*-axis as polar axis and the *x*-axis as azimuthal axis. As we are interested in the boundaries of the angular region into which the electrons emit radiation, we only consider the deflection of the electrons from their initial propagation direction $\delta \zeta ={\zeta }_{i}-\zeta$ with $\zeta \in [\theta ,\varphi ]$ and where the electrons’ initial propagation direction in the considered setup is given by ${\theta }_{i}={\varphi }_{i}=\pi \mathrm{/2}$. It can be shown that in the *ϕ*-*θ* plane the emission is confined to a rectangular emission box in the plane spanned by the emission angles $(\theta ,\phi )\in ([{\phi }_{i}-|\delta \phi ({t}_{0})|,{\phi }_{i}+|\delta \phi ({t}_{0})|],[{\theta }_{i}-\delta \theta ({t}_{0}),{\theta }_{i}]))$, where *t*_0_ is the time at which the angular deviations are maximal (s. Methods). The azimuthal deflection *δϕ* points into both halfspaces *δϕ* > 0 and *δϕ* < 0, symmetrically. The polar deflection *δθ* in a plane wave, on the other hand, always points towards the laser’s propagation direction, resulting in *δθ* > 0. The advantage of analyzing the emission box rather than only one of the angles is that the emission box is sensitive to the electron dynamics in a broader parameter range than just one of the angles. For a laser pulse with a typical Gaussian shape in time $g(\eta )=\exp [\,-\,2{(\eta /{\tilde{\tau }}_{L})}^{2}]$ with the physical and scaled FWHM pulse durations *τ*_*L*_ and ${\tilde{\tau }}_{L}={\tau }_{L}/\sqrt{\log \,\mathrm{(2)}}$, respectively, an electron bunch with constant energy ${\varepsilon }_{i}$ yields ${t}_{0}\equiv 0$, i.e., emission towards the maximal emission angles originates from the regions of highest field strength, i.e., the pulse’s temporal and spatial center. For an electron bunch with a linear energy chirp, on the other hand, due to the time dependence of the electrons’ energy, contrary to the naive guess, *t*_0_ instead increases for increasing pulse durations *τ*_*L*_. Consequently, as $\delta \phi ({t}_{0}),\delta \theta ({t}_{0})$ explicitly depend on *τ*_*L*_, the emission box’s boundaries facilitate to determine the laser’s pulse duration. To quantitatively exemplify the dependency of the emission box’s boundary angles on *τ*_*L*_ we consider the interaction of a moderately relativistic electron bunch ${\varepsilon }_{i}/m{c}^{2}=10$. We assume the bunch to have a large energy spread $\Delta \varepsilon ={\varepsilon }_{i}$ and to be short ${\tau }_{E}=55$ fs, as can be typical for an electron bunch from a laser-accelerator not optimized for monochromaticity^38,39,48^. The laser is assumed to have a dimensionless amplitude of *ξ* = 10 at a central frequency $\hslash {\omega }_{0}=1.55$ eV. For these parameters quantum electrodynamical (QED) effects like single-photon recoil of the electron are suppressed by a factor $\chi ={\varepsilon }_{i}\xi \hslash {\omega }_{0}/{m}^{2}{c}^{4} \sim {10}^{-4}$ ^35,49^ and are hence neglected. Plotting the resulting changes of the emission box’s cutoff angles (s. Methods) we find a clear dependence on the laser’s pulse duration (s. Fig. 2). Thus, determining the boundary angles of the emission box emitted by a chirped electron bunch scattering from a relativistically intense laser pulse allows to determine the pulse’s duration.

## Numerical Simulation

**Figure 3:**
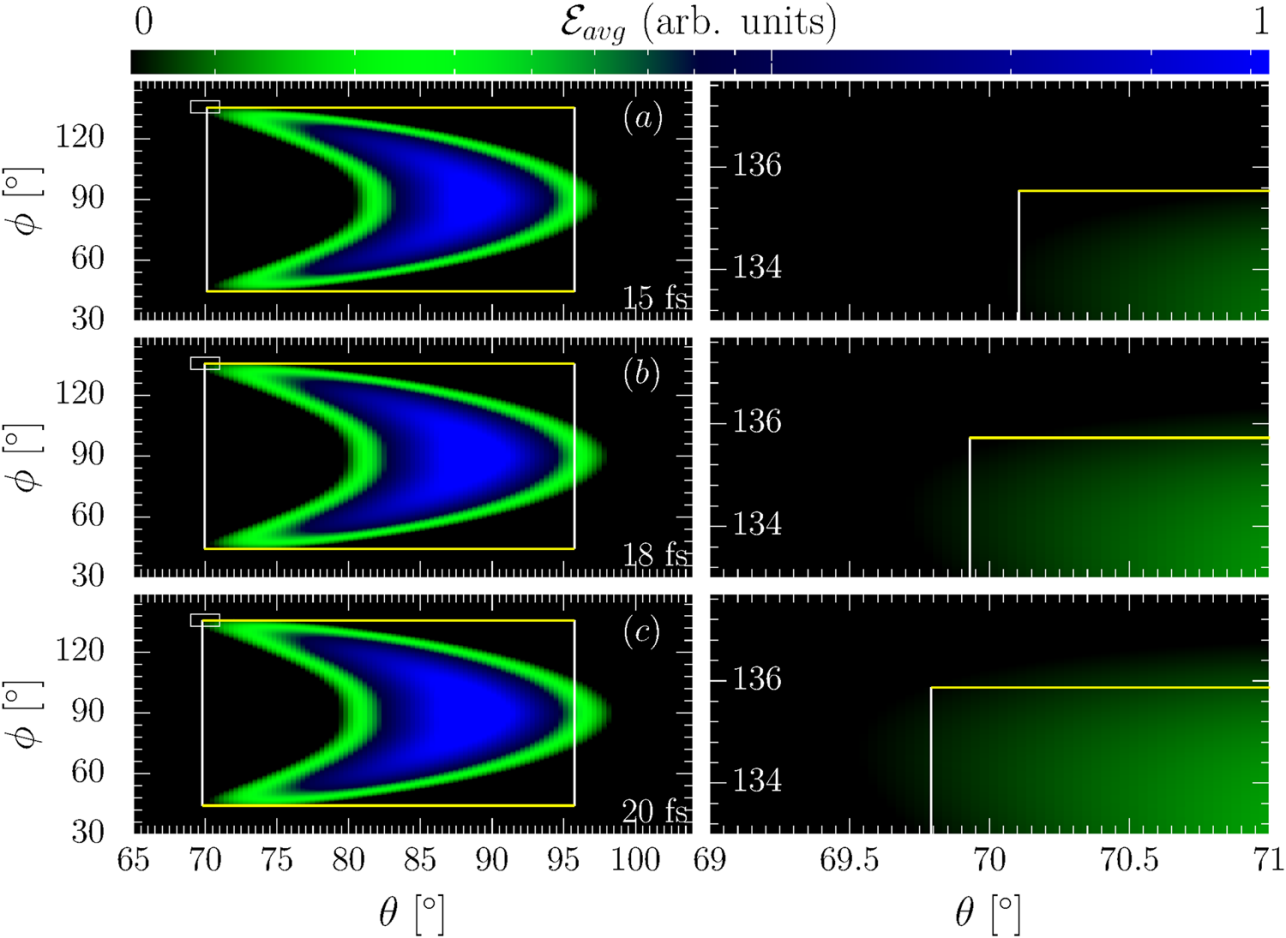
Left: Numerically computed angular radiation distributions from a chirped electron bunch (parameters in the text) for different pulse durations (bottom right corners) with the analytical emission box superimposed (large white rectangles). Right: Zoom-in on the radiation pattern’s cutoff region (small inlay rectangles in left column). The colorbar represents the average energy emitted per electron (${ {\mathcal E} }_{avg}$) in arbitrary units.

To corroborate the above conclusions, we performed a full numerical simulation of the above scattering (s. Methods). We model a realistic electron bunch with Gaussian density distribution in its transversal dimension (FWHM width *R* = 3 *μ*m) and uniform in longitudinal direction, containing 300 numerical particles. The laser is modeled as a Gaussian laser focus including non-paraxial correction terms up to ${\mathscr{O}}({\varepsilon }^{5})$ in the small focusing parameter $\varepsilon ={\lambda }_{0}/\pi {w}_{0}$, containing non-transverse laser field components, as derived in^50^. We consider a laser of not too tight focusing to a spot size of ${w}_{0}=10\,\mu$m. For this focusing it was shown that high-order beyond-paraxial effects do not significantly affect particle dynamics inside the laser focus^51^. Furthermore, the work done by the laser’s longitudinal electric field is expected to be small in the here studied perpendicular collision geometry as, e.g., compared to a head-on collision geometry, since the electrons spend only very little time in the laser focus where such components are significant. From Larmor’s formula one can estimate each electron to emit 1–10 photons in the scattering, whence a sufficient radiation signal would still be obtainable from a low density bunch, which can be used to mitigate space charge effects. We checked that for an electron bunch of less than 10^6^ particles its space charge does not cause significant longitudinal spreading compared to its ballistic expansion resulting from a large energy spread as assumed above. The bunch’s spectral broadening due to space charge was experimentally found to be significantly smaller than $\Delta \varepsilon /{\varepsilon }_{i}=1$ even for much denser electron beams^40,52^. Superimposing the analytically predicted emission box over the numerically obtained radiation signal of the total bunch divided by the number of particles in the bunch, i.e., the average energy per solid angle element ${ {\mathcal E} }_{avg}$ emitted from a single electron in the bunch, scattered from laser pulses of various durations we find an excellent reproduction of the numerically obtained emission box boundaries by the analytical prediction (s. Fig. 3). We stress that the exact numerical evaluation of the Liénart-Wiechert potentials corresponds to the particle dynamics found from solving the equations of motion for each numerical particle in the laser focus exactly. The finite size of each particle’s emission cone is hence taken into account exactly, in contrast to the analytical model where we only estimated its effect to be of order $\Delta \theta  \sim {5}^{\circ }$. From the observation that the analytical model still agrees with the numerically found emission patterns over a broad range of parameters we infer that the electrons’ angular emission patterns are dominated by their trajectory and the emission cone’s finite size is only a small perturbation, as we conjectured based on the analytical model. Hence, with a proper detector calibration, or gauge point, for the emission box, relative changes of the detected radiation emission pattern’s boundary box can be realistically interpreted. Such a gauge point can be ideally inferred from the emission patterns off an unchirped electron bunch, as for this bunch the emission box is unique and can be used to infer the cutoff intensity at its corresponding boundaries. Relative changes to this unchirped emission box then encode information on the pulse duration, as demonstrated in the numerical example. We wish to stress that while the emitted energy is normalized to its maximal value, this maximal value is chosen the same in all plots shown in each figure, whence all numerical results are directly comparable. We furthermore highlight that the numerically obtained emission spectra are spectrally integrated, whereas resolving the emission patterns spectrally would yield valuable additional information: Frequencies *ω* much smaller than the critical frequency ${\omega }_{c}:\,=\,3{\varepsilon }_{i}^{3}/{m}^{3}{c}^{5}\rho$, where *ρ* is the electron trajectory’s radius of curvature, are emitted into a wide angle range $\Delta {\theta }_{{\rm{low}}} \sim m{c}^{2}{({\omega }_{c}/\omega )}^{\mathrm{1/3}}/{\varepsilon }_{i}$, whereas for $\omega \gg {\omega }_{c}$ the angle range is much narrower $\Delta {\theta }_{{\rm{high}}} \sim m{c}^{2}{({\omega }_{c}\mathrm{/3}\omega )}^{\mathrm{1/2}}/{\varepsilon }_{i}$^53^. The intricacies of studying spectrally resolved signals, however, have to be left for future studies for the sake of conciseness. We thus conclude that the predicted boundary angles of the emission box are defined by emissions close to the laser focus, where the employed plane wave model is a good approximation for the electron dynamics. We note, that contrary to the analytical prediction, there occurs emission into angles $\theta  > {90}^{\circ }$, which we attribute to ponderomotive push from the laser focus, not accounted for in the model. This push, due to the spatial gradient of the intensity profile divided by the scattered particle’s mass and the laser’s frequency squared, can be estimated to cause an effect always smaller than the peak field’s square divided by the laser’s spot size, the scattered particle’s mass and the laser’s frequency squared. Then, the momentum imparted by the ponderomotive force onto a relativistic electron with a Lorentz factor $\gamma ={\varepsilon }_{i}/m{c}^{2}$ can be estimated to be ${p}_{{\rm{pond}}}\lesssim mc{\xi }^{2}\mathrm{/4}\gamma$. The momentum imparted onto an electron by the laser field, on the other hand, is given by ${p}^{\perp } \sim mc\xi$, ${p}^{\parallel } \sim mc{\xi }^{2}\mathrm{/2}\gamma$^54,55^ in the direction along the laser’s polarization and propagation direction, respectively. Consequently, we see that, even without assuming an exact analytical model of the laser envelope’s spatial gradients the presented simple estimate already shows that the momentum due to ponderomotive scattering cannot exceed the momentum gained from the laser momentum in the parameter regime $\xi  \sim {\varepsilon }_{i}/m{c}^{2}$. The resulting angular deflection thus satisfies $\Delta {\theta }_{{\rm{pond}}}\lesssim m{c}^{2}{\xi }^{2}{\mathrm{/10}}^{2}{\varepsilon }_{i}$. Shifting the emission box boundary from *θ* = 90° to $\theta ={90}^{\circ }+{180}^{\circ }/\pi (m{c}^{2}{\xi }^{2}{\mathrm{/10}}^{2}{\varepsilon }_{i})$ in Fig. 3, we find this estimate approximately satisfied also numerically. Towards all other boundary angles of the emission box, direct laser acceleration dominates over the ponderomotive push. We conclude that detecting the emission box cutoff angles for *ϕ* and *θ* < 90° gives a reliable determination scheme for the laser’s pulse duration.

## Experimental Implementation

An experimental realization of the outlined direct pulse duration determination can be envisaged in analogy to the successful experimental determination of an ultra-intense laser pulse’s intensity directly in its focus^37^, which, however, was incapable of providing dynamic information about the laser pulse. In this experiment, a laser-accelerated electron bunch was brought back into collision with the laser pulse used for its acceleration and the electrons’ emission recorded. This had the advantage, that the laser-accelerated electrons are intrinsically synchronized with the laser pulse. On the other hand, even without intrinsic synchronization with the laser pulse one can gauge the experiment by recording the shots’ total emitted intensity, mapping the achieved overlap between the electron bunch and laser pulse^56^. In this section we now wish to pointwise delineate the weaknesses and error sources possibly affecting an according realization of the proposed pulse duration method:

1. Extending the concept of ^37^ to allow for a pulse duration measurement requires to impart an energy chirp onto the electron bunch, which can be achieved by optimizing the electron bunch to feature a large energy spread and let it propagate through free space for an appropriate amount of time. While this may lead to a nonlinear energy chirp of the electron bunch, contrary to the linear chirp assumed in the analytical model (s. Methods), the precise analytical shape of the energy chirp does not need to be known exactly, but only the bunch’s overall energy variation over its length, i.e., an “averaged chirp”. To corroborate this statement we varied the exact analytical form of the electron bunch’s energy distribution from linear, as used in the above theory, (s. Fig. 4(b)) to the nonlinear distribution $\varepsilon (t)={\varepsilon }_{i}(1-4|t|t/{\tilde{\tau }}_{E}^{2})$ with the same energy spread and bunch duration (s. Fig. 4(a)) and compared the spatial distributions of the radiation emitted in the collision of these two bunches with laser pulses of the various durations considered in the above numerical example, i.e., 15, 18 and 20 fs. We find the two radiation patterns from the nonlinear bunch chirp to be virtually indistinguishable, provided the energy spread and bunch length are equal. We thus assert that, in order to make the suggested pulse duration scheme feasible, instead of the exact form of the chirp it is enough to determine the bunch’s “averaged chirp”, which can be inferred from the bunch’s energy spread and duration. Consequently, the electron bunch’s duration, energy spread, and central energy suffice as measurement parameters for implementing the proposed pulse duration measurement scheme. These parameters can be obtained by spectroscopically recording the electron bunch after the interaction, when it is no longer needed. We have thus repeated the above numerical simulations including the energy loss of the electrons due to the emission of radiation in the form of radiation reaction, by replacing the Lorentz force equation by the Landau-Lifshitz equation for computing their dynamics^57,58^.

**Figure 4:**
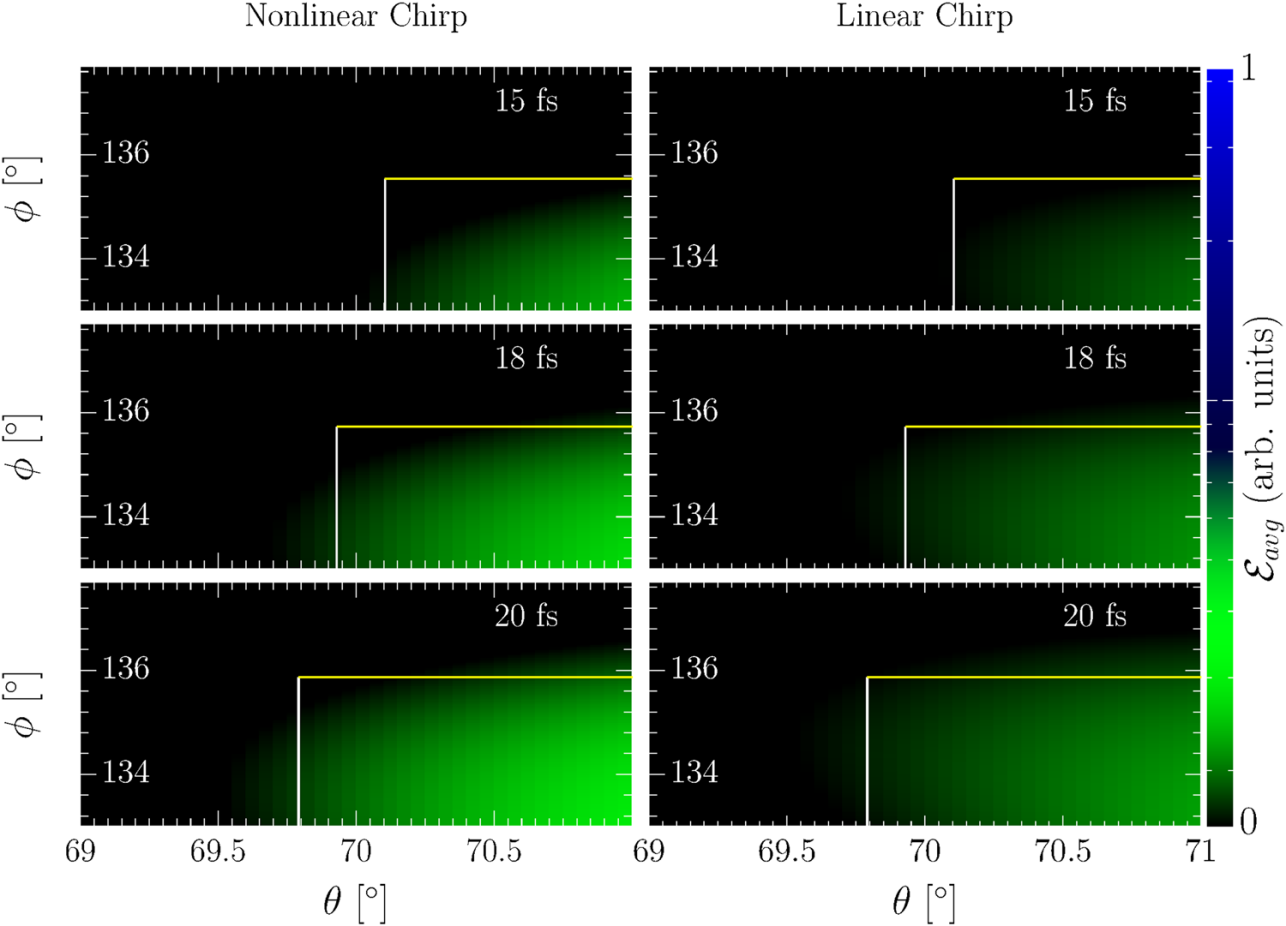
Left: Numerically computed angular radiation distributions from a nonlinearly chirped electron bunch (parameters in the text) for different pulse durations (top right corners) with the analytical emission box superimposed (large white rectangles). Right: Comparison to angular emission patterns from a linearly chirped bunch with equivalent energy spread. The colorbar represents the average energy emitted per electron (${ {\mathcal E} }_{avg}$) in arbitrary units.
  We found the difference in the radiation signal to be negligible, whence the spectral bunch properties before and after interaction with the laser pulse are comparable, facilitating a post-scattering characterization of the electron bunch.
2. Furthermore, uncertainty in the pulse duration determination from measuring the emission box’s cutoff angles may arise from improperly known parameters of the electron bunch, such as its bunch width or central energy which can be determined to an accuracy of $\Delta {\varepsilon }_{i}\lesssim \mathrm{1 \% }$^52,59–61^. The impact of these uncertainties can be quantified analytically within our plane wave model by a standard error propagation. The error in the angle measurement due to uncertainties in the electron energy and energy spread is computed as $\Delta {\zeta }^{{\varepsilon }_{i}}\approx \Delta {\varepsilon }_{i}(d\zeta /d{\varepsilon }_{i})$ and $\Delta {\zeta }^{\varDelta \varepsilon }\approx \Delta (\Delta \varepsilon )(d\zeta /d\Delta \varepsilon )$, respectively, where $\zeta \in [\theta ,\phi ]$. For laser-accelerated electron beams of MeV energies energy measurements with resolutions as low as 0.3% were reported^52^. Using this optimal resolution we find the uncertainties in the angle measurement to be given by $\Delta {\theta }^{{\varepsilon }_{i}}\approx {0.08}^{\circ }$, $\Delta {\phi }^{{\varepsilon }_{i}}\approx {0.09}^{\circ }$, $\Delta {\theta }^{\Delta \varepsilon }\approx {0.08}^{\circ }$ and $\Delta {\phi }^{\Delta \varepsilon }\approx {0.09}^{\circ }$. In a real experiment, however, the cutoff angles of the emission box can be detected only to a certain accuracy, experimentally reported to be of the order $\Delta \theta =\Delta \phi ={0.1}^{\circ }$ ^37^, comparable but still larger than the uncertainty due to errors in the energy measurement. The error in the pulse duration measurement caused by this inaccuracy in the angle measurement can be derived through an error propagation to be $\Delta {\tau }_{L}^{\zeta }\approx \Delta \zeta (d{\tau }_{L}/d\delta \zeta )$ with $\zeta \in [\theta ,\phi ]$. From Eqs. (4a,b) we find complex expressions for the derivatives which we do not need to report here. Evaluating these expressions for the above studied parameters we find the error of the pulse duration to be comparable for both *θ* and *ϕ* and to be negligible for pulse durations above 18 fs (s. Fig. 5). Moreover we find the error to decrease for longer pulses, as a fixed change in ${\tau }_{L}$ leads to increased changes in *δϕ* and *δθ*.

**Figure 5:**
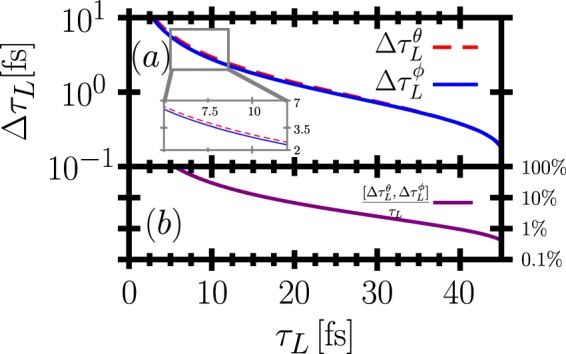
(**a**) Absolute errors of the pulse duration measurement as a function of pulse duration resulting from a 0.1° inaccuracy of both cutoff-angle measurements. (**b**) Relative error resulting from the maximum absolute error.
3. In contrast to these analytical estimates, effects of uncertainties in the bunch’s geometric length and width cannot be assessed in our analytical model, as this is based on the assumption of a plane wave laser. We have thus performed numerical simulations varying the width of the electron bunch by more than 50%, to account for beam stretching, and its length by more than 30%, as the beam spreading in longitudinal direction is less pronounced than transversely. Neither of these scans resulted in significant changes of the radiation patterns (s. Fig. 6).

**Figure 6:**
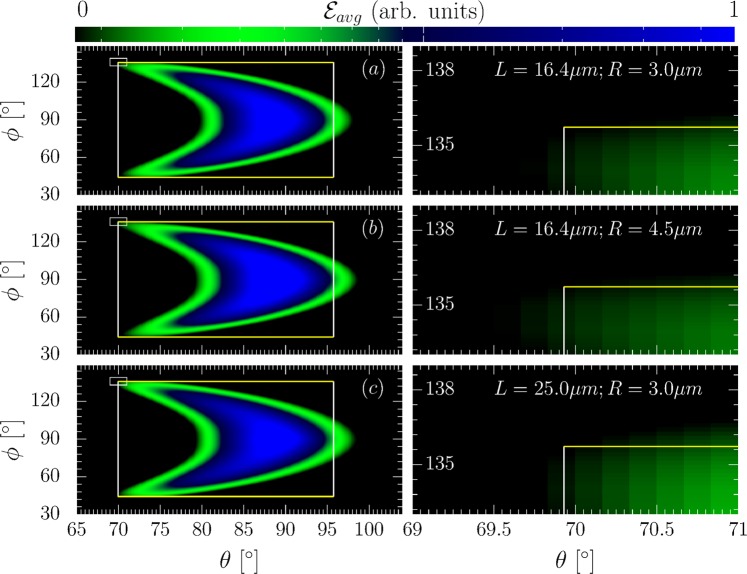
Left: Numerically computed angular radiation distributions from a electron bunches with varying length and width (parameters in right column) for ${\tau }_{L}=18$ fs with the analytical emission box superimposed (large white rectangles). Right: Zoom-in on the radiation pattern’s cutoff region (small inlay rectangles in left column). The colorbar represents the average energy emitted per electron (${ {\mathcal E} }_{avg}$) in arbitrary units.
4. Equally, a realistic electron bunch will have a finite divergence, typically on the order of mrad, which the analytical model cannot include. We have thus repeated the above simulations with an electron bunch of 1 mrad divergence and also found the resulting changes in the radiation patterns (s. Fig. 7) to be insignificant with respect to the above case (s. Fig. 3). We additionally note that an experimental electron beam pointing instability of order 1 mrad hence will also not mask the dependence on the pulse duration, since a divergent electron bunch encompasses all possible alterations of the bunch’s propagation directions due to such an instability.

**Figure 7:**
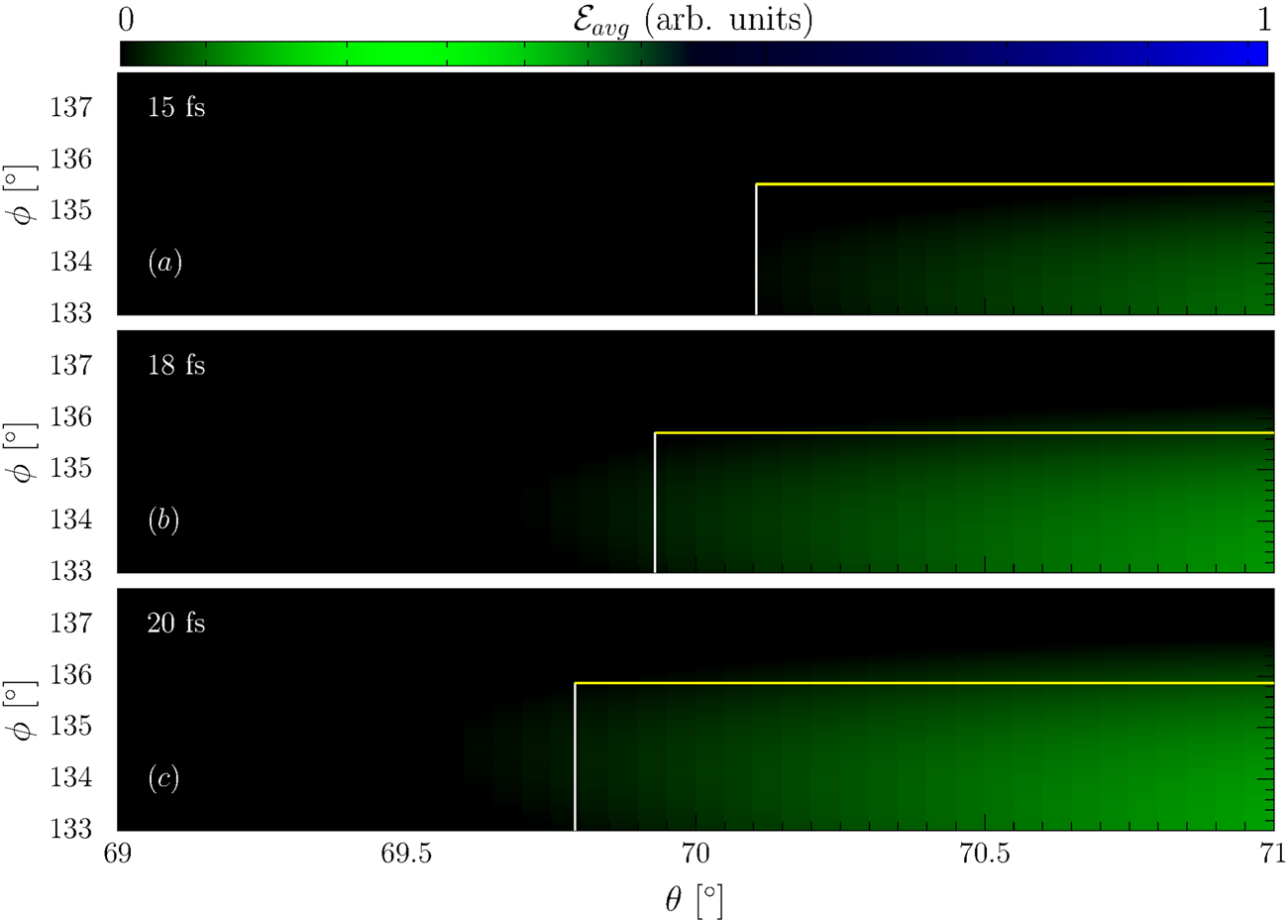
Numerically computed angular radiation distributions with 1 mrad divergence of the electron bunch. The bunch and laser parameters are discussed in the text. The colorbar represents the average energy emitted per electron (${ {\mathcal E} }_{avg}$) in arbitrary units.
5. Additionally, we note that while the envelope of an ultra-short laser pulse is usually well modeled by a transform-limited Gaussian^7,8,12,62,63^, it is a priori not known. To check the influence of a non-Gaussian envelope we repeated the numerical simulations for a super-Gaussian envelope function $g(\eta )=1.035\exp [\,-\,\mathrm{8(}\eta /{\tilde{\tau }}_{L}{)}^{4}]$, resembling a flat-top pulse of the same FWHM pulse duration more strongly deviating from common ultra-short laser pulse shapes than to be expected at most laser facilities. The changed numerical factors with respect to the chosen Gaussian pulse shape ensure that in both cases ${\tau }_{L}$ denotes the laser intensity profile’s (square of field profile) FWHM duration and that the integral over the super-Gaussian intensity profile is equal to that over the Gaussian intensity profile, indicating that the total energy contained in the pulse is conserved. Even with this strongly deviating pulse profile, we found the cutoff angles of the numerically simulated radiation signal still to be well described by the analytical formula for a Gaussian pulse, provided the FWHM durations of the two pulse profiles were equal (s. Fig. 8). Physically, this independence of the signal on the pulse profile can be explained by the fact that the energy of the electrons in the bunch changes independently of the pulse profile. Consequently, the ratio of the electrons’ energy to the laser field at the time instants $t=\pm \,{\tau }_{L}\mathrm{/2}$ is the same for both pulse profiles. Since the parameter ${\tau }_{L}$ is determined through measuring this ratio which is encoded in the emission pattern, however, the precise pulse profile does not influence the proposed determination scheme.

**Figure 8:**
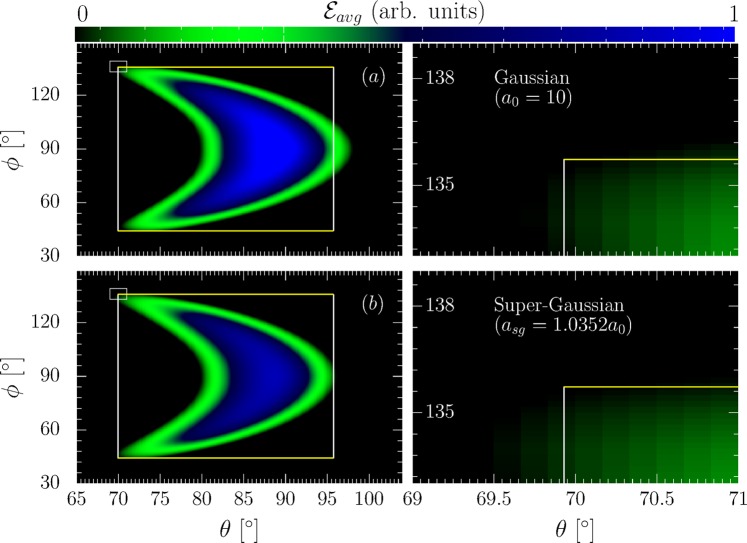
Left: Numerically computed angular radiation distributions for a Gaussian (**a**) and super-Gaussian (**b**) temporal laser pulse profile with the analytical emission box superimposed (large white rectangles). Right: Zoom-in on the radiation pattern’s cutoff region (small inlay rectangles in left column). The FWHM pulse duration for either case is considered to be ${\tau }_{L}=18$ fs. The bunch length and radius is considered to be 16.4 and 3 *μm* respectively. The colorbar represents the average energy emitted per electron (${ {\mathcal E} }_{avg}$) in arbitrary units.
6. Furthermore, the good agreement of the emission box with Eq. (4) indicates that the proposed scheme is insensitive to the pulse’s structure at sub-maximal intensities. The reason for this insensitivity of the presented scheme to pulse shape variations is that its signal are the boundaries of the emission region, corresponding to the maximum deflection of the electrons inside the laser field. This maximal deflection occurs only at the regions of highest laser field strength, whence the presented method is robust against changes in the laser’s temporal structure at sub-maximal intensity values, e.g., due to the presence of a pre-pulse, as they are by definition of too low intensity to trigger the electrons into emitting significant radiation.
7. Next, we wish to estimate the effect of the electrons’ finite sized emission cone: Emission into large angles *δθ* with respect to a relativistic electron’s instantaneous propagation direction drops approximately as^53^
  3 $$\frac{dI}{d\Omega } \sim {\frac{dI}{d\Omega }|}_{\delta \theta =0}\,\exp [-3\frac{\omega {\gamma }^{2}\delta {\theta }^{2}}{{\omega }_{c}},]$$
  translating in the above studied numerical example to a emission cone with opening angle $\Delta \theta  \sim m{c}^{2}/{\varepsilon }_{i} \sim {5}^{\circ }$. On the other hand, from Eq. (2) we infer that for the studied parameters ${\varepsilon }_{i}=\xi m{c}^{2}$ the electrons’ total emission pattern is angularly confined to a wide cone of opening angle $\Delta \zeta  \sim {45}^{\circ }$, indicating that the opening angle of the instantaneous emission cone can maximally amount to a relative error of $\Delta \theta /\Delta \zeta  \sim \mathrm{9 \% }$. On the other hand, the absolute size of the emission cone of 5° would result in prohibitively large accuracy loss for the pulse duration determination, if it were a random error source. However, this is not the case, as the angular radiation distribution is deterministic according to Eq. (3), and defining a threshold intensity $d{I}_{{\rm{th}}}=dI(\delta {\theta }^{\ast })$ at which we identify the according exponential drop-off with the cutoff angles of Eq. (4) one can see that a change $\delta {\vartheta }$ in the electron’s propagation direction, amounting to the replacement $\theta \to \theta -\delta {\vartheta }$, results in the threshold intensity being reached at the same $\delta {\theta }^{\ast }$, provided the intensity in forward direction ${dI|}_{\delta \theta =0}$ is conserved. This condition, however, is automatically fulfilled, if the electron’s energy is conserved and its trajectory merely deflected, as is the case in the proposed setup. Hence, we predict that if we consider relative changes of the cutoff-angles, as we implicitly do by comparing all emission patterns with the same intensity normalization, neglecting the electrons’ finite emission cone size still results in a reliable prediction for the laser pulse duration. This conjecture is further confirmed by the agreement between the analytical model with the (properly normalized) exact numerical simulations of the emitted radiation signal, which take into account finite Δ*θ*-effects.
8. Finally, we note that spatiotemporal coupling of the laser pulse may distort its spatial and temporal structure in a complicated mixed fashion^64,65^. It is challenging to provide a general quantitative model for such nonlinear effects. However, as demonstrated above, structural changes in the laser’s precise temporal shape, as may be a result of spatiotemporal couplings, do not crucially affect the proposed scheme, which was demonstrated to provide unique information on the former’s temporal FWHM. Due to spatiotemporal couplings, however, this quantity might change throughout the laser focus, as a result of a spatial dependence of the laser’s temporal structure. From Eqs. (4, 5) we infer that, as the proposed method is mainly sensitive to the maximal emission angles, is likely to provide a signal corresponding to the maximally achieved laser pulse duration. Hence, one could view the pulse duration derived from the proposed method as an upper limit, which might be underrun in parts of the laser focus, due to spatiotemporal coupling. Furthermore, uncertainties in the laser’s peak intensity, as can additionally result from spatiotemporal couplings would affect the proposed scheme only as an overall factor in the size of the emission box and not the relative angle changes.

## Conclusion

We have put forward a scheme capable of determining the duration of a laser pulse of in principle arbitrarily high intensity. It takes advantage of the fact that the electromagnetic radiation emitted by an electron interacting with an ultra-intense laser pulse is angularly confined to an emission box, whose size depends on the ratio of the electron’s and laser’s local energy and field strength, respectively. Thus, we demonstrated that by imposing a temporal energy chirp onto an electron bunch it is possible to imprint information about the laser’s temporal structure onto the frequency integrated emission signal. This information notably also contains a clear dependence on the pulse’s temporal duration, which can henceforth be measured. Assuming an angular detection accuracy of 0.1° we gave a quantitative example of a pulse duration determination with an order-of-magnitude accuracy for pulses with durations of order ${\tau }_{L} \sim {\mathscr{O}}(10)$ fs.

## Methods

### Analytical derivations

The electrons’ deflection angles are given by the inverse tangents of the following ratios of the electrons’ instantaneous momentum components $\delta \phi ={\rm{atan}}({p}_{x}/{p}_{y})$, $\delta \theta ={\rm{atan}}({p}_{\perp }/{p}_{z}\,)$, where ${p}_{\perp }=\sqrt{{p}_{x}^{2}+{p}_{y}^{2}}$ is the electron’s momentum perpendicular to the polar axis. From the analytically known expressions for an electron trajectory inside a plane wave^54,55^ it can be derived that for the suggested perpendicular collision geometry these maximal deflection angles are given by

4a $$|\delta \phi ({t}_{0})|={\rm{atan}}(\frac{mc\xi g({t}_{0})}{{p}_{i}({t}_{0})})$$

4b $$\delta \theta ({t}_{0})={\rm{atan}}(\frac{{m}^{2}{c}^{3}{\xi }^{2}{g}^{2}({t}_{0})}{2{\varepsilon }_{i}({t}_{0})\sqrt{{p}_{i}^{2}({t}_{0})+{(mc\xi g({t}_{0}))}^{2}}}),$$

where ${p}_{i}(t)=|{{\boldsymbol{p}}}_{i}(t)|$ and *t*_0_ is the time at which *δϕ*, *δθ* are maximal. In order to determine this time for the chosen Gaussian temporal pulse shape we neglect the field’s oscillatory structure, and compute the time at which Eq. (4) are extremal. For an electron bunch with constant momentum ${p}_{i}(t)={\rm{const}}\mathrm{}.$ this is simply ${t}_{0}\equiv 0$. For an electron bunch with a nontrivial time-dependence of *p*_*i*_(*t*), however, the ratio $m{c}^{2}\xi /{\varepsilon }_{i}$ changes over time in addition to *g*(*t*). Naturally, as the electron bunch propagates perpendicularly to the laser pulse, there will be a different emission box for each point in space. However, we are only interested in the boundaries of the total emission box, originating from the point of strongest electron deflection in the center of the laser focus. At this point the electrons’ energy is a function of time only, which we approximate as changing linearly according to $\varepsilon (t)={\varepsilon }_{i}(1-2t/{\tilde{\tau }}_{E})$, where we defined the scaled electron pulse duration ${\tilde{\tau }}_{E}:={\tau }_{E}{\varepsilon }_{i}/\Delta \varepsilon$ with the bunch’s FWHM duration ${\tau }_{E}$ and its energy spread Δ*ε* and all other definitions as before. The negative sign for the chirp term ensures the electrons’ energy to decrease over time, as is realistic if the chirp is envisaged to originate from free-space propagation of an electron bunch with a large energy spread. Furthermore, it leads to an increase of the emission angles with time, avoiding the emission box’s boundaries to be covered by radiation emitted at earlier times. Using this model for the electron bunch chirp we find

5 $${t}_{0}=\frac{1}{2}({\tilde{\tau }}_{E}-\sqrt{{\tilde{\tau }}_{E}^{2}-{\tilde{\tau }}_{L}^{2}})\mathrm{}.$$

A real solution ${t}_{0}\in {\mathbb{R}}$ only exists provided ${\tilde{\tau }}_{L}\le {\tilde{\tau }}_{E}$, indicating that for too long laser pulses the emission angles do not reach a maximum value but change monotonically over the whole interaction time.

### Numerical particle dynamics

The trajectories of the electrons in the bunch are evaluated by solving the relativistic Lorentz equation of motion for the charge particles in an external electromagnetic fields i.e. $d{p}^{\mu }/dt=q{F}^{\mu \nu }{u}_{\nu }$. The time dependent laser fields (${F}^{\mu \nu }$) are calculated according to a Gaussian focus model including non-paraxial correction terms up to ${\mathscr{O}}({\varepsilon }^{5})$ in the small focusing parameter $\varepsilon ={\lambda }_{0}/\pi {w}_{0}$^50^. The effect of radiation reaction is included in the electron dynamics via the perturbative approximations of the Landau Lifshitz force equation^57,58^. It is found that for the fields and energies studied in this work radiation reaction does not play a significant role. The initial position of the electron bunch is chosen such that the central part of the chirped electron bunch would interact with the laser at its peak intensity.

### Numerical radiation signal

Once the trajectories of the electrons are calculated, the the power radiated by the bunch is computed by integrating the Liénart-Wiechert potentials^51,53,66^ over the particles’ trajectories. The contribution of all the electrons in a bunch is summed over for the angular window in *θ* and *ϕ* shown in the numerical figures.

### Algorithm

The particles are pushed using the standard Boris leapfrog algorithm, wherein the particle motion is decomposed into the motion in the electric and magnetic field separately and hence the **v** × **B** rotation is properly achieved^67^. The numerical stability of the Boris algorithm manifests in its widespread usage in the state of the art particle-in-cell simulations.

## Data Availability

All relevant numerical data supporting our findings are available from the corresponding author upon reasonable request.
